# The Alabama Dental Association

**Published:** 1893-08

**Authors:** J. M. Walker


### PROCEEDINGS.

### The Alabama Dental Association.

Reported for THE DENTAL REGISTER by Mrs. J. M. Walker.

The Alabama Dental Association held its twenty-fourth annual meeting in the parlors of the Caldwell Hotel, Birmingham, April 11, 12, 13, 14, 1893.

Dr. C. L. Boyd (Eufaula) the President, occupied the chair.

The meeting was opened with prayer by the Rev. T. J. Beard, rector of the Church of the Advent.

After the usual addresses of welcome, on behalf of the city and of the resident dentists, the President delivered the

### ANNUAL ADDRESS.

He dwelt briefly upon the elevated standing attained by the dental profession, and the benefits it confers upon humanity, and the unrevealed possibilities of Preventive Dentistry through the proper education of the people, especially mothers, in a comprehension of the demands of nature in the development and preservation of the teeth. He also spoke of the too common violations of the dental law by men who are both unqualified and unworthy to bear the name of dentist, and urged the importance of having such parties brought to account, and the law, as enacted, enforced.

Dr. E. S. Chisholm spoke of the great difficulty the Board has experienced in getting at the facts, and asked the co-operation of the members of the Association in furnishing the Board with information in the premises.

The scientific not being prepared to report, at the first session, "Incidents of Office Practice" were related and discussed. Several cases of accidental injuries, resulting in fractured maxillæ, necrosis, etc., were detailed, with mode of treatment; several cases of so-called "cancer" cured by proper treatment of dental lesions, were described and discussed.

Dr. A. A. Eubank had cured a case in which an alveolar abscess from a devitalized superior central incisor was discharging opposite the root of a second bicuspid with a clear tract from one opening to the other, the incisor being the only dead tooth in the mouth.

Dr. W. G. Browne (Atlanta, Ga.) described a method of making gold plate or bridgework without swaging or the use of dies. No. 30 soft pure gold is burnished directly upon the plaster cast, and the piece stiffened with platinum wire soldered around the borders, enough solder being used to make a smooth, level surface. In one case, constructed in this manner, the six anterior superior teeth were standing in the mouth but so badly worn down that Richmond Crowns were put on the incisors and gold crowns on the canines. Over the latter a second crown was telescoped, attached to each one of which was a soft gold saddle bearing four posterior teeth on either side of the mouth. These saddles were not connected in the anterior portion of the mouth, but were held in position by a narrow bar across the rear portion of the roof of the mouth, the whole being stiffened with platinum wire. The teeth were attached with rubber in the usual manner. Marble dust and plaster, half and half, are used for the cast. The teeth on the saddles, in this case, antagonize with strong lower molars, the bite also being raised in crowning the incisors.

Dr. E. S. Chisholm corroborated his testimony, given on previous occasions, in favor of the "Marshall Anchor Denture," which he has inserted in a large number of cases, giving great satisfaction. This plate offers all the advantages of high-priced bridgework, with almost the cheapness of an ordinary rubber denture.

Dr. R. C. Young described a very peculiar case. A central incisor having a devitalized pulp, from a blow, was opened from the palatine surface and treated several times with carbolic acid on cotton, left in the root canal. After the third treatment the boy failed to return. A year later he came for other work, saying that "the other tooth was all right." Having left only cotton in the root canal he examined it, but could not find any trace of the opening made the year previous; absolutely nothing to show that it had ever been drilled into.

Dr. H. C. Boyd described his method of using the English pinless teeth, a tooth of very fine material, but a cheap tooth, because of having no platinum pins. He uses gold wire in the holes of the teeth, allowing the ends to project sufficiently to solder on a continuous band, investing in plaster and asbestos for soldering. This makes a strong but not cumbersome piece, being waxed up, invested and vulcanized as usual.

Dr. T. M. Allen, at the first night session, exhibited diagrams of teeth with very crooked root canals, eliciting an interesting discussion of the best modes of procedure in cleaning out and filling such roots, the materials employed, what to do with broken off drills and broaches, etc. The method advocated by the majority of the speakers was: *first find the canal* with a fine broach; then enlarge it cautiously with graduated Gates-Glidden drills, raising and pushing the drill alternately to be sure that it is not caught, depending upon the patient to let you know when you have *got there*. Forcing the medicaments into contact with living tissue outside of the apex, and out through the fistulous tract, if open, was advised by several speakers. Others were very cautious to avoid going through the apex. A probable second canal in the anterior root of first molars is often the unsuspected cause of subsequent trouble. When the drill accidentally goes through the side wall of a crooked root, as sometimes happens.

Dr. Boyd advises flooding the canal with thin chloro-percha, pumping it in until it goes out through the side.

For filling root canals, Dr. A. A. Pearson uses paste made of oxide of zinc and oil of cloves, into which he works a little of the alloy used in amalgam fillings.

Dr. W. G. Browne uses oxychloride of zinc because of its mummifying action on any shreds of organic matter that might be left in the canal. He uses the hot-air-cavity dryer to dry the canal thoroughly, and fills the root canal with a copper point softened by heat sufficiently to conform to the shape of the canal.

Dr. E. S. Chisholm thinks that if the apex is hermetically sealed, and the mouth of the canal and the crown cavity perfectly filled, it is immaterial whether there is anything more than a column of air in the intervening space. The elements of disease coming from the outside, if both doors are hermetically sealed, nothing injurious can enter.

Dr. Boyd thinks that moisture would find its way through the pericementum and the dentinal tubuli. Dr. Boyd saturates the canal with carbolic acid, after removing all debris and washing out with peroxide of hydrogen. He absorbs all excess of carbolic acid with a little cotton on a broach, dipping this moistened cotton in iodoform with which he dusts the walls of the root canal and the cavity, filling with chloro-percha pumped in. Dr. Boyd believes that by his methods 95 per cent. of all root canals can be opened up to and through the apex, and 90 per cent. filled clear to the apex.

Dr. Wm. Crenshaw (Atlanta, Ga.) fills with very thin oxychloride of zinc, imbedding in it a fine copper or gold wire, or more preferably a gutta-percha point as avoiding the risk of a metal point projecting through the apex.

Dr. T. M. Allen believes, as the result of many trials, that the very large majority of teeth supposed to have their roots "filled to the apex" are not so filled, and that splitting open three or four such teeth, after extraction, will prove this to be so. He said "we think we fill them, but we don't do it."

Under the head of Physiology, the subject of "Nutrition" was discussed at some length.

Dr. C. A. Merril attributes the decay of the teeth of women and children to their large use of starchy foods, bread and butter, etc. The dentist, to be worthy of the title of doctor or teacher, should instruct his patients, especially mothers and children, in regard to dental hygiene and the importance of proper diet.

Dr. R. R. Freeman (Nashville, Tenn.) dwelt at some length on the motto, "clean teeth don't decay," which he inscribes on his bill-heads as a rebuke to any question as to the amount of the bill. He also ascribes much of the trouble with the teeth of young persons to modern methods of education, the brain being cultivated at the expense of the physique. Instead of being educated—drawn out—they are depressed and crushed by the burdens laid upon them.

Dr. Chisholm thinks that too much stress is laid upon the matter of diet; occupation, climate, modes of life, etc., all having their modifying influence.

Dr. Westmoreland (Columbus, Miss.) attributes the imperfections in children's teeth more largely to eruptive diseases than to any other factor. With the idea that children "get over" measles, chickenpox, etc., more easily when young, they are exposed to them just when the teeth are at the most critical stage of formation, and he believes this to be largely the source of the poor teeth we have to deal with.

Dr. J. Y. Crawford (Nashville, Tenn.) attributes the specific affections of the oral cavity to want of functional activity. The peculiar influences of our highly civilized life lessen the functional activity of tooth structure, and result in lesions of the entire organism. We work the brain until it outstrips the body, resulting in an impaired, overtaxed nervous system. The teeth are prematurely erupted while imperfectly developed. The roots are prematurely absorbed, the deciduous teeth are prematurely removed, and the permanent teeth are prematurely erupted. Being brought into contact with external influence while yet imperfectly developed, they, in their turn, are subject to early decay. A child has no business at hard study till nature has provided it with a perfect apparatus with which to build up brain and body. Do not wean a child till it has an unbroken row of teeth. Do not put it to hard study till it has an unbroken row of permanent teeth. Nature will tell you when to put your child to study, and when he is fit for it.

In the discussion of "Pathology and Surgery," Dr. L. G. Noel gave the history of a case of diseased antrum with an abscess over the bicuspid roots. From constitutional vice the bones were all affected, a portion of the septum nasi having been lost. She was dismissed as cured after a long course of treatment, but shortly after fell down stairs and fractured the lower jaw, necessitating an interdental splint. It is not considered probable that she will ever recover, owing to the impairment of the osseous system.

Dr. J. Y. Crawford deprecated over-treatment of the antrum, being specially emphatic in the reiteration of *let it alone.*

Dr. W. G. Browne gave the history of an interesting case. The deciduous teeth were knocked out by a blow, resulting in diseased tissues and the eruption of permanent incisors so badly decayed as to necessitate a bridge at the age of fourteen years.

Dr. Geo. Eubank had a case in which, by a kick from a horse, a section of the lower jaw, carrying the six anterior teeth, was broken loose at both ends and driven back in the mouth half an inch. A jackscrew was inserted between the bicuspids, driving them apart until the broken part was placed in position, where it remained held firmly in place by natural forces without any splint.

Dr. Browne had a case in which the jaw was broken at the ramus, also by a kick from a horse. After the bone had reunited the wisdom tooth erupted pointing into the soft tissues of the cheek. Every effort was made to extract it but it could not be moved. It will probably have to be sawed out.

Dr. C. V. Rosser reported a case in which an abscess from a second bicuspid on the buccal surface failed to heal, after being lanced. Dr. Rosser being absent from the city for several months, during his absence the bicuspid was extracted " by advice of her physician," and the abscess lanced again, on the palatine surface, without healing. On examination Dr. Rosser found another opening an inch back, at the junction of the hard and soft palates with free passage from one to the other, and considerable necrosed bone, a piece the size of the thumb nail, and several other smaller pieces of the palatal bone being removed by him, it healed in about thirty days, leaving a slightly thickened membrane from front to rear.

Dr. R. C. Young spoke of the great value of *pyrozone*, which he said "acts like magic." Dr. Young reported a case which he had had under treatment. A child of eight years of age, whose surroundings in every way, both social and sanitary, had been of the very best, and the child in perfect health and the teeth all sound. There was a fistulous opening over both deciduous canines with a clear tract from one to the other, through which the probe or medicaments passed freely from one to the other. The permanent canine, which was plainly perceptible just above the deciduous tooth, erupted immediately after the extraction of the latter. Both of the deciduous cuspids were extracted, and were found to have live nerves and unabsorbed roots. There was evidently necrosed bone between the two openings.

Dr. J. Y. Crawford expressed the opinion that necrotic action was undoubtedly superinduced by pus burrowing from one point to the other, the primary cause of irritation being the migration of the permanent tooth from a proper to an improper position, pressure upon the unabsorbed root of the deciduous tooth causing traumatic action in the inflammation and abscess. Absorption of the roots of the deciduous teeth, though a physiological function, is an extraordinary function, and as such, more susceptible than those which run *pari passu* with existence.

Dr. E. S. Chisholm gave the history of an interesting case of hemorrhagic diathesis, in a child two years of age. In running about with a pencil in its mouth the child fell lacerating the roof of the mouth. For four days every effort was made by two prominent physicians to arrest the hemorrhage, even applying the galvanic cautery. They gave the child up to die, as it was vomiting blood, and the system so depleted that they pronounced recovery impossible. It occurred to the parents that a dentist often had to check hemorrhage in the mouth and Dr. Chisholm was called to see the child. He found it lying in the mother's lap, death hourly expected. He at once constructed an appliance of wire to retain a compress, passing a wire between the incisors and throwing out a loop to the rear, beyond the point of laceration, with a cross wire from the first molars just erupted, cutting retaining grooves in the teeth. This served to hold closely in position a soft compress of lint, saturated with Monsel's solution, which at once arrested the hemorrhage and the child recovered. A cure of several cases of "cancer," so pronounced by attendant physicians, was related, the "cancers" proving to be simple dental lesions—an abscess from a dead tooth—a broken root left under the plate, or an imbedded wisdom tooth.

A striking case of malpractice was narrated by Dr. Merril. An abscess from a badly decayed wisdom tooth was treated for months by two physicians successively, by poulticing and lancing from the outside on the cheek and under the chin, a cure being finally effected by the simple removal of the offending wisdom tooth. The patient was so reduced by lack of nourishment and the drain upon his system from the long continued suppuration that he was confined to the house for ten weeks, and it was several months before he recoverd his usual health.

The subject of "Pyorrhea Alveolaris" was discussed at considerable length, especially its etiology.

Dr. J. Y. Crawford made emphatic the statement that "we don't cure disease." We only bind up the parts and nature works the cure. In so-called Pyorrhea Alveolaris, the parts involved are not inclined to reproduce themselves. The gum-tissue is semi-cartilaginous, and does not respond to treatment; the alveolar process does not reproduce itself. You may be able to assist nature by eliminating causes, and mechanically tighten the teeth, and perhaps even cure isolated spots, but we must work upon the expectant method, meeting indications as they arise, mitigating conditions. It is a self-limited disease if the patient has the vitality to withstand the onslaught. It is not *per se* local; there is a vitiated condition of the secretions from constitutional impression. As to the *cause*, there is a long list of influences and factors which lead up to and produce it, which we do not understand. Dr. Crawford considers *mal-occlusion* a most potent factor, dating away back in the history of the individual. The baby molars having been allowed to decay at three, four, five years of age, the surroundings are in a state of violent inflammation while the permanent teeth are in the stage of upbuilding and growth. By virtue of this long continued inflammation, the bone around the permanent teeth is diseased, resulting in disarrangemant of the gums. In the healing of the soft tissues, cicatricial tissue forms, interfering with the eruption of the permanent teeth, with consequent irregularity, mal-occlusion and pyorrhea alveolaris.

Dr. Boyd believes the disease to be purely local and due solely to lack of cleanliness, and lack of proper exercise of the surrounding tissues; not encouraging the teeth to grow out of the jaw a sufficient length to give them space or allow the lower teeth to go inside without being crowded and jammed together. Exercise is as necessary for the gums and the bony tissues around the teeth as for other portions of the body. *Massage* is the proper treatment for the gums, rubbing them well inside and out, night and morning. Encourage children to do this regularly, and provide them with such food as requires exercise of the jaws and teeth in mastication. This will develop the jaw and make the teeth strong and their surroundings healthy. In this way we can avoid the direct causes of decay and pyorrhea alveolaris.

Dr. R. C. Young.—Pyorrhea Alveolaris is a local manifestation of a systemic disease. It is never cured by the dentist. It sometimes gets well, but not until after the teeth are lost. After the socket has been destroyed it is not reproduced any more than an arm. The disease is contagious and may be transferred by the instruments of the dentist.

Dr. Wm. Crenshaw said that in his own experience he had not found mal-occlusion to be a factor in producing pyorrhea. On the contrary some of the worst cases he had known had the teeth in perfect occlusion, as in his own mouth and that of his father. Both have perfect occlusion, and both are victims of this destructive pericementitis.

Dr. Boyd said that his own mouth offers an exaggerated case of mal-occlusion, but without any tendency to pyorrhea or accumulations of tartar.

Dr. Crenshaw, in the treatment of this disease uses bichloride of mercury 1 to 1600, or 4 grains to the fluid lb. as a mouth wash. He considers this strength perfectly safe, even if swallowed by the teaspoonful. It is the most effectual gargle and wash in diphtheria, acting also as a perfect preventive, if used in time. This solution, if used on the tooth brush, will kill the tendency to pyorrhea alveolaris. Absolute cleanliness is the only treatment of any avail.

Dr. E. S. Chisholm is satisfied that the disease is hereditary. The deposit in true pyorrhea is at the end of the root, and is a secretion from the devitalized tissues around the apex.

Dr. L. G. Noel regards lowered vitality as the most potent factor in the production of this disease.

Dr. Crawford thinks that if the disease were hereditary as claimed it would sometimes be seen in children with the deciduous teeth. They have soft, cheesy tartar, and they have soft, chalky teeth that decay easily, but they never have pyorrhea. Disease is not inherited—only a susceptibility to disease—a dyscrasia of the blood.

Dr. Freeman thought that in any case we should treat it as if we expected to cure it, and we may carry the patient along in comparative comfort for many years. There is some relief in the mere hope of cure. Never destroy the teeth except as the last resource. Wire them up, supporting them by their stronger neighbors, and keep the surroundings sweet and clean, and you will have done much to mitigate suffering, even if you do not absolutely cure the disease.

Dr. L. G. Noel says it is a disease of adolescence, pointing to lowered vitality. Children do not have it because when the alveolar process is forming the bones are in active life; there is high vascularity of the tissues; rich blood is being poured in. Pyorrhea begins after the system has entered upon its downward career.

Dr. Crawford spoke at some length on the subject of the World's Columbian Dental Congress—its aims and objects, and the means for accomplishing them. He spoke particularly of the prize medal offered for the best elucidation of the means of caring for the teeth of the poor, and especially of the children of the poor—a question that demands the attention of the civilized world. In all great national crises the lower classes form the bone and sinew of the country. Laws are made for treating and eliminating the diseases of hogs and cows, and horses and fowls, but nothing is done for the children of the people.

Dr. R. C. Young read a paper entitled "The Carbo-Hydrates as a Factor in Tooth Decay."

He took the ground that while bacteria take a very active part in the destructive work of caries, in themselves they are powerless. Their great ally is Hydrogen in combination. There can be no decay without acids, and no acid has any affinity for the lime salts of the teeth but those containing hydrogen, the most prolific source of acids in the mouth is the fermentation of the carbo-hydrates or starch-containing foods. This fermentation is produced by bacteria. Saliva added to starch or sugar and kept at blood temperature will become acid in twenty-four hours. Sterilize the starch and we still have the acid reaction. Sterilize the saliva and we have no such reaction. The starch food is first converted by the ptyalin of the saliva into grape sugar, and this in turn is separated into lactic acid by the bacteria; the acid unites with the lime in the enamel of the teeth, forming lactate of lime. The softened layer of enamel under the microscope is found to consist of enamel rods or prisms and bacilli. When, by this process, the mineral matter of the tooth is dissolved out, the albuminous portion of the tooth is peptonized or digested out by animalculæ. By this alternate action the entire organ may be destroyed unless interfered with by the processes of dental surgery. These facts give us a clew how to hold the disease in check. By mechanical and chemical means we can keep the mouth *caeteris paribus*, in such a state that much can be done to overcome deleterious environments.

This subject was discussed at some length by Drs. Chisholm, Noel, Pearson, Browne, Crawford, Young and others.

Dr. Noel said that in 1874 he read a paper before the American Dental Association in regard to the molecular reaction of saliva on cooked starch, in the formation of lactic and acetic acid. In another paper read, much later, at Old Point, and another at Niagara, he had taken the same position, endeavoring to show the analogy between caries and digestion; caries being a dissolving out of the limesalts from the animal matrix of the tooth. Dr. Wm. H. Atkinson criticised his position severely. Dr. Geo. Watt subsequently wrote to him: "My boy, you're right."

He said he does not believe that bacteria cause pus formation, but we find them wherever fermentation is going on, because there they find something to feed upon. He believes their function is wholly catalytic. Caries spreads, as shown by Dr. Miller, by the proliferation of microscopic organisms which crowd into the tubuli of the dentine. The result is the same whether due to catalytic action or the pressure of increasing numbers breaking down the walls of the tubuli.

Dr. Chisholm thinks that the action of acids being all sufficient for the work of destruction, and that as we cannot exclude bacteria, which are found everywhere, it is sufficient for us to devote ourselves to the work of counteracting the acid conditions. To comprehend the subject of bacterial action requires the work of a lifetime in microscopical studies. We must get in our protective and preventive work before the bacteria begin, and that gives us enough to do.

Dr. W. G. Browne spoke of the sudden inroads of rapid white decay sometimes observed in mouths previously in good condition, saying that he had learned, from repeated observation, to regard this as the precursor of very serious illness if not of death. In people who have been overworked, he feels persuaded that this condition means dissolution.

Dr. R. C. Young attributes it to the work of uric acid. He considers the condition of the teeth an important factor in diagnosis, and predicts that in ten years from now a physician will as invariably consider the condition of the mouth and teeth as he does the pulse, in determining the general condition.

Dr. J. Y. Crawford defines caries as a chemico-vital process, the result of an acid condition of the saliva, in which the most prominent factors are lactic and acetic acids. He said that his hobby, if he had one, is the importance of the recognition of the condition of the oral cavity as a factor in disease. If we can satisfactorily solve the problem of dental caries and how to prevent it, it will be the stepping stone toward the comprehension of the wonderful phenomena of disease in the human system. We need to look more deeply into pathological conditions and define more clearly the etiology of dental lesions. The dentist should stand on the same platform as the doctor, for he is a healer—not a mechanic. His practice imposes upon him equally high responsibilities.

The subject of "Ethics" was discussed by Drs. Rousseau, Crawford, and Chisholm.

Dr. Crawford said that in view of the coming World's Dental Congress, it is most timely now to study up the written law of ethics, as the great majority do not comprehend all that it implies. The code of ethics is designed for the protection of both the public and the profession; it is not more in the interests of one than of the other, and it is essential that the true intent of the code be thoroughly comprehended. Its laws are immutable; they are fixed by a higher power, and based upon charity and common sense. Dr. Crawford condemned with great severity the endorsement of patent nostrums by ministers of the gospel, who, in lending their signatures, and in some instances, even portraits to such endorsement do more positive harm than can be counterbalanced by the good done in a lifetime. It weakens their influence, impairs the confidence of thinking men and paralyzes their power for good.

Dr. E. S. Chisholm gave an instructive talk on "Operative Dentistry," taking for his text twelve head lines, which he had placed upon the blackboard, as follows:

CHISHOLM'S METHOD.

1. Excavate all decay.
2. Trim all sharp edges.
3. Secure three retaining points.
4. Smear the surface of cavity with cement of creamy consistency.
5. Anchor crystalloid gold, before cement hardens.
6. Project amply beyond cervical walls.
7. Build on this with cohesive gold.
8. Condense with small instruments against the walls.
9. Build out to full contour.
10. Cautiously key the gold.
11. Dress filling down to cavity border.
12. Dress down and burnish again.

Dr. Chisholm said that the subject of filling teeth is of the most practical importance to the dentist, and occupies fully three-fourths of his time. The most complicated and difficult are those involved in the preservation of the incisor teeth, on account of the very thin walls left when the intervening dentine has been destroyed by decay. As the experience of many years he has reduced the matter to the above definite system, as the most reliable method of preserving the form, color and strength of these most essential teeth, and of preventing the recurrence of decay. Dr. Chisholm then proceeded to enlarge upon each of the points involved in his "method," especially in regard to the use of cement as a lining for all cavities in the anterior teeth where gold is used as the filling material. The coating of cement must be very light; only sufficient to disguise the color of gold through thin walls, and to hold in place the crystalloid gold used as a foundation upon which to build out the contour with cohesive foil. He likens this foundation to bricks set in mortar by a builder. The cement must not be allowed to reach the margins of a cavity, but must be confined to the bottom where moisture cannot reach it, and the crystalloid gold imbedded in it before it hardens. Such a filling will last a lifetime. He said that he does not offer this as a fancy or a theory. It has stood in his hands the test of years.

Dr. H. D. Boyd said in the discussion of the subject, that he was perhaps extreme in his appreciation of the cements. He uses cement to save the tooth, and covers with gold to save the cement. His only criticism on Dr. Chisholm's method would be that he uses too little cement. Instead of a "mere film" of cement, he would say a "mere veneer of gold."

Dr. R. R. Freeman thought it possible that in using cement as thin as described, the large proportion of phosphoric acid might endanger the pulp, though he had never had any bad re-results from his own use of the cements, both oxychloride and oxyphosphate. For the posterior teeth he uses tin, especially at the cervical margins, being convinced that it is a conservator of tooth structure. If adjoining proximal cavities, run down deep under the gum margins, lay a strip of tin foil across from one cavity to the other; put your matrix down over that, and carry the tin well down over the margins of tooth cavities. Then with cotton, by Herbst's rotary motion, force the tin down to the cervical margin. Remove the cotton and you may fill the rest of the cavity with cement and it will never wash out, and the teeth will not decay any further.

Dr. R. C. Young said that the cemented in inlays had given him an idea which he had found of practical value in certain cases. When a tooth is exceedingly sensitive and the patient very nervous, in those cases, where it is next to impossible to obtain proper retaining points or undercuts, shape the cavity very simply, saucer shaped if you can do no better; insert a well condensed, well polished gold filling. Then push it out, and after washing both the cavity and the filling (or inlay, as it will be), with absolute alcohol, cement it in again. This was done the first time as the result of an accident, and he expected to refill the tooth the next day, but it seemed so solid that he concluded to let it rest awhile and watch the result. It proved so satisfactory that he has adopted it as a method of dealing with supersensitive teeth for very nervous patients.

In the order of merit, as tooth filling material, Dr. Crawford places gold first, tin second, cement third and last. For amalgam, he finds no place at all. With gold, tin and cement, the skillful operator can save every human tooth that is savable. Use gold, as a rule, in all suitably located cavities in teeth of good structure. Use tin when decay is so located that we may look for recurrence through contact of those vitiated fluids which are a factor in decay. Use the cements temporarily in teeth of poor, soft structure.

In the discussion of "Dental Education and Literature," Dr. J. T. Stewart spoke at some length on the importance and necessity of instructing patients at the chair. Train them to rely on your *judgment* as to what is best for the case in hand. Reply to their questions, be explicit with them and arouse their understanding.

Dr. T. M. Allen insisted strongly on the necessity of the more thorough preliminary education of dental students. The standard for graduation has been raised through the efforts of State Examining Boards, but it is equally important that the standard for admission be raised. All of the time of the three years' course is needed for the education of the student in dental science. The colleges can not give an elementary education also. Dr. Allen also condemned severely the harsh dealing with which papers read before Associations are met.

A young man is invited and urged to prepare a paper for his State Society. He spends time and thought upon it, but instead of his efforts meeting with a degree of appreciation, he is made to feel that he has only made a fool of himself. He is disheartened and discouraged and determines that his first paper shall be his last, and perhaps withdraws from society work altogether. If a young man offers us a paper, pick out its best points; point out his errors gently, but be lenient with its possible defects, encourage his efforts and he may in time furnish valuable papers.

Dr. Chisholm: Dr. Allen has expressed it exactly. Old as I am I never come before you with a paper without expecting to have it torn to pieces, and this seems to be done with a spirit which disgusts and discourages a man. We show no quarter.

Dr. Chisholm then gave a history of the work done by the State Dental Examining Board, of which he has been a member for twelve years, except for one year when he was President of the State Society. He spoke of the great advances made along the line, especially in the character of those who become students.

One great drawback to the profession is that it is the chosen line of the great majority of that unfortunate class of young men who are "poor but respectable"—who are too poor to acquire an education, and too "respectable" to do the work that can be done without an education. They look upon honest labor as dishonorable or dishonoring, and think they see a chance to live without labor, in dentistry, the cheapest and easiest and quickest occupation they can get into to make money. They are so destitute—they make such frantic appeals—that there seems to be no way to deny them. And so they are admitted to the dental college, and they manage somehow to work their way through, believing that the little they know is all they will ever need.

We object to having men come among us who are not thoroughly prepared to do honor to themselves and to us, and we have found it necessary to require even holders of diplomas to submit to rigid examination. We feel no opposition to the young men who come before us. We want their good will. We almost violate the law ourselves in giving permits, etc., to encourage them.

Dr. T. M. Allen: The Board never rejects a man who is up to the standard. It is not the fault of the Board if a man falls below it.

PROSTHETIC DENTISTRY.

Dr. J. Y. Crawford spoke regretting the disposition apparent to relegate mechanical dentistry to the background as if beneath our attention. But he said the best dentist is the all-round dentist. The one who is able and ready to do whatever is best for each and every one of his patients. He should be competent and willing, if necessary, to not only fill teeth but to clean teeth, to extract teeth, to construct artificial dentures, or to correct irregularities. To be a dentist is not to be *a plugger, a puller, or a cleaner.* There is often great responsibility attached to the proper construction of an artificial denture; it may help to prolong the life of an individual, and it is as much our duty to use our skill in this as in making fine contour fillings.

For a lower denture Dr. Crawford described the method of swaging between Babbitts metal dies a piece of thick aluminum plate with rubber attachments, which, he says, will give the greatest possible satisfaction.

Dr. T. M. Allen described the old fashioned, but still the best, way of making a gold plate with sand mold, and zinc and lead die and counter die.

Dr. W. G. Browne described his greatly simplified method of making partial gold plates, using No. 30 gauge pure gold, which he burnishes directly upon the plaster cast, using marble dust and plaster, half and half, for the model. He strengthens and stiffens the piece by soldering platinum wire all around it, using enough solder to make an even surface. He described a piece recently made, for which he placed Richmond crowns on the four superior incisors with gold crowns on the cuspids. On each side he telescoped a second removable crown over the cuspid, attaching a saddle bridge carrying four posterior teeth. The saddles were connected by a band one-half inch wide of gold across the roof of the mouth at the posterior portion of the saddles. No dies were used, the gold being burnished on to the plaster models, and stiffened with platinum wire across the band and around the edge of the saddle bridges. The piece is removable by the patient, and is worn with great satisfaction.*

Another piece made after the same plan was a partial lower plate, with the right anterior teeth in position. In this piece the gold was carried across the anterior teeth, a double plate being used, stiffened with triangular bits of platinum between the two plates, the inner piece being not quite so broad as the one next to the teeth ; solder was used only at the points where the platinum was inserted, allowing the rubber covering the lingual surface of the piece to flow between the two thicknesses of gold, giving a strong attachment.

Dr. Crawford thought this method only applicable to a collar or band for a single tooth. For in gold plate swaging between metal dies is the only way to secure perfect accuracy.

Dr. R. R. Freeman advocates the reliable old fashioned die

* Two weeks later, at the meeting of the Georgia State Dental Society, Dr. Browne exhibited this piece in the mouth of the patient.—Reporter.

and counter die of zinc and lead. He said it is a well known fact that the expansion of plaster and the contraction of zinc mutually counteract each other, giving a most accurate fit. He said "do not follow blindly after any one, but think, and give a reason for the *think* that is within you."

Dr. Chisholm uses type metal as giving the most clear-cut die, with lead for the counter die.

To get an accurate adjustment of a Logan crown, Dr. R. C. Young takes an impression of the exposed end of the root after it has been approximately trimmed, and makes a cast. He then smokes the cast, and on applying the crown you see exactly where to grind.

Dr. Freeman paints the end of the root red. The pigment adhering to the crown shows where to touch it up with much less trouble than making a cast.

Dr. Boyd uses a little rouge and glycerine for the same purpose.

This was the last subject discussed.

The election of officers resulted as follows:

Dr. T. M. Allen, Eufaula, President; Dr. R. A. Rush, Selma, First Vice-President; Dr. W. E. Proctor, Sheffield, Second Vice-President; Dr. S. W. Foster, Decatur, re-elected Secretary; Dr. C. M. Rousseau, Montgomery, re-elected Treasurer.

Troy was selected as the next place of meeting, the time being the second Tuesday in April, 1894.

On motion, adjourned.

---

CATERPILLARS IN PILL-BOXES.—Mr. E. B. Boulton, F.R.S., fascinated the Biology Section of the British Association with the results of his experiments on caterpillars hatching in pill-boxes. The pepper moth was the particular insect which he experimented on, and his experiments show that if you take an egg of one of these and grow it in a gilded pill-box you get a golden caterpillar. Again, if the pill-box be black, so is the caterpillar; while a mixed environment produced a muddled creature, just as in man the environment of the slum or the palace pretty much determines a person's characteristics.

